# History of infertility, risk of type 2 diabetes and HbA_1c_ levels in the Nurses’ Health Study II

**DOI:** 10.1007/s00125-026-06784-5

**Published:** 2026-06-30

**Authors:** Leslie V. Farland, William J. Degnan, Siwen Wang, Audrey J. Gaskins, Jorge E. Chavarro, Janet W. Rich-Edwards, Deirdre K. Tobias, Stacey A. Missmer

**Affiliations:** 1 Department of Epidemiology and Biostatistics, Mel and Enid Zuckerman College of Public Health, University of Arizona, Tucson, AZ USA; 2 Department of Obstetrics and Gynecology, College of Medicine-Tucson, University of Arizona, Tucson, AZ USA; 3 Department of Nutrition, Harvard T.H. Chan School of Public Health, Boston, MA USA; 4 Department of Epidemiology, Rollins School of Public Health, Emory University, Atlanta, GA USA; 5 Channing Division of Network Medicine, Brigham and Women’s Hospital, Boston, MA USA; 6 Department of Epidemiology, Harvard T.H. Chan School of Public Health, Boston, MA USA; 7 Division of Women’s Health, Department of Medicine, Brigham and Women’s Hospital and Harvard Medical School, Boston, MA USA; 8 Division of Preventative Medicine, Department of Medicine, Brigham and Women’s Hospital and Harvard Medical School, Boston, MA USA; 9 Department of Obstetrics, Gynecology, and Reproductive Biology, College of Human Medicine, Michigan State University, Grand Rapids, MI USA; 10 Department of Obstetrics and Gynecology, University of Michigan, Ann Arbor, MI USA

## Abstract

**Aims/hypothesis:**

The aim of the study was to investigate the relationship between infertility history, the risk of type 2 diabetes and HbA_1c_ levels.

**Methods:**

Among the participants in the Nurses’ Health Study II (*n*=116,429), those who reported infertility (>12 months of trying to conceive), both overall and by infertility diagnosis, were compared with gravid women with no history of infertility. We used Cox proportional hazard models to estimate the HR and 95% CI of type 2 diabetes and adjusted for a priori potential confounding variables. Person-time was stratified a priori by age (≤50 and >50 years); in secondary analyses, we restricted the sample to those with a BMI of <25 kg/m^2^ at 18 years of age. We used linear regression models to assess the prospective association between the history of infertility and log-transformed blood HbA_1c_ concentrations measured on average at age 44, among a subset of participants with this measure (*n*=2399).

**Results:**

The association between a history of infertility and the risk of type 2 diabetes differed before and after the age of 50 (*p* value, test for interaction: 0.0004). Up to 50 years of age, infertility history was associated with a 32% greater risk of type 2 diabetes (95% CI 1.22, 1.43); for specific infertility diagnoses, ovulatory disorders were associated with a 67% greater risk (95% CI 1.48, 1.89), whereas tubal blockage (HR 1.29 [95% CI 1.01, 1.64]), male factor infertility (HR 1.30 [95% CI 1.08, 1.57]) and ‘cause not found’ (HR 1.26 [95% CI 1.05, 1.51]) were all associated with a ~30% greater risk of type 2 diabetes. After the age of 50, the association between overall infertility and type 2 diabetes attenuated (HR 1.12 [95% CI 1.06, 1.19]), as did associations between ovulatory disorders and type 2 diabetes (HR 1.18 [95% CI 1.05, 1.32]). The association between tubal blockage and the risk of type 2 diabetes remained similar after the age of 50 (HR 1.26 [95% CI 1.05, 1.51]). The overall association between infertility and the risk of type 2 diabetes remained elevated among women with a BMI of <25 kg/m^2^ at the age of 18 (≤50 years, HR 1.32 [95% CI 1.20, 1.44]; >50 years, HR 1.11 [95% CI 1.04, 1.19]). Women with a history of infertility also had slightly higher mean HbA_1c_ levels than women without a history of infertility (relative difference: 0.7% [95% CI 0.04, 1.32]), reflecting a minimal absolute difference in HbA_1c_.

**Conclusions/interpretation:**

Women with a history of infertility had a greater risk of developing type 2 diabetes, particularly prior to the age of 51 years. Specific reasons for infertility were associated with an elevated type 2 diabetes risk, including ovulatory disorders and tubal factors. These associations were independent of BMI.

**Graphical Abstract:**

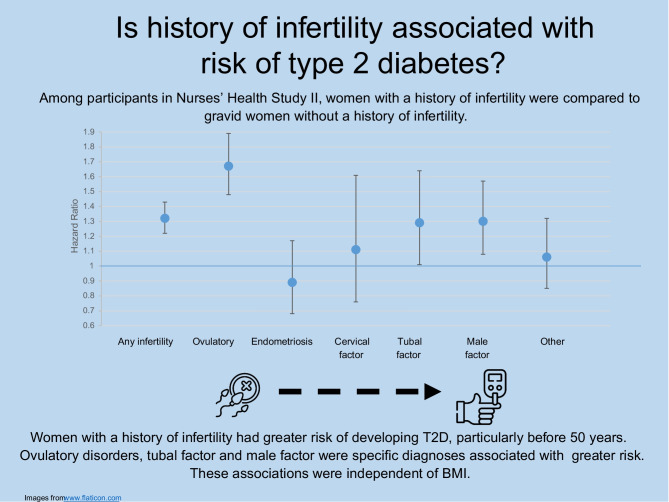

**Supplementary Information:**

The online version contains peer-reviewed but unedited supplementary material available at 10.1007/s00125-026-06784-5.


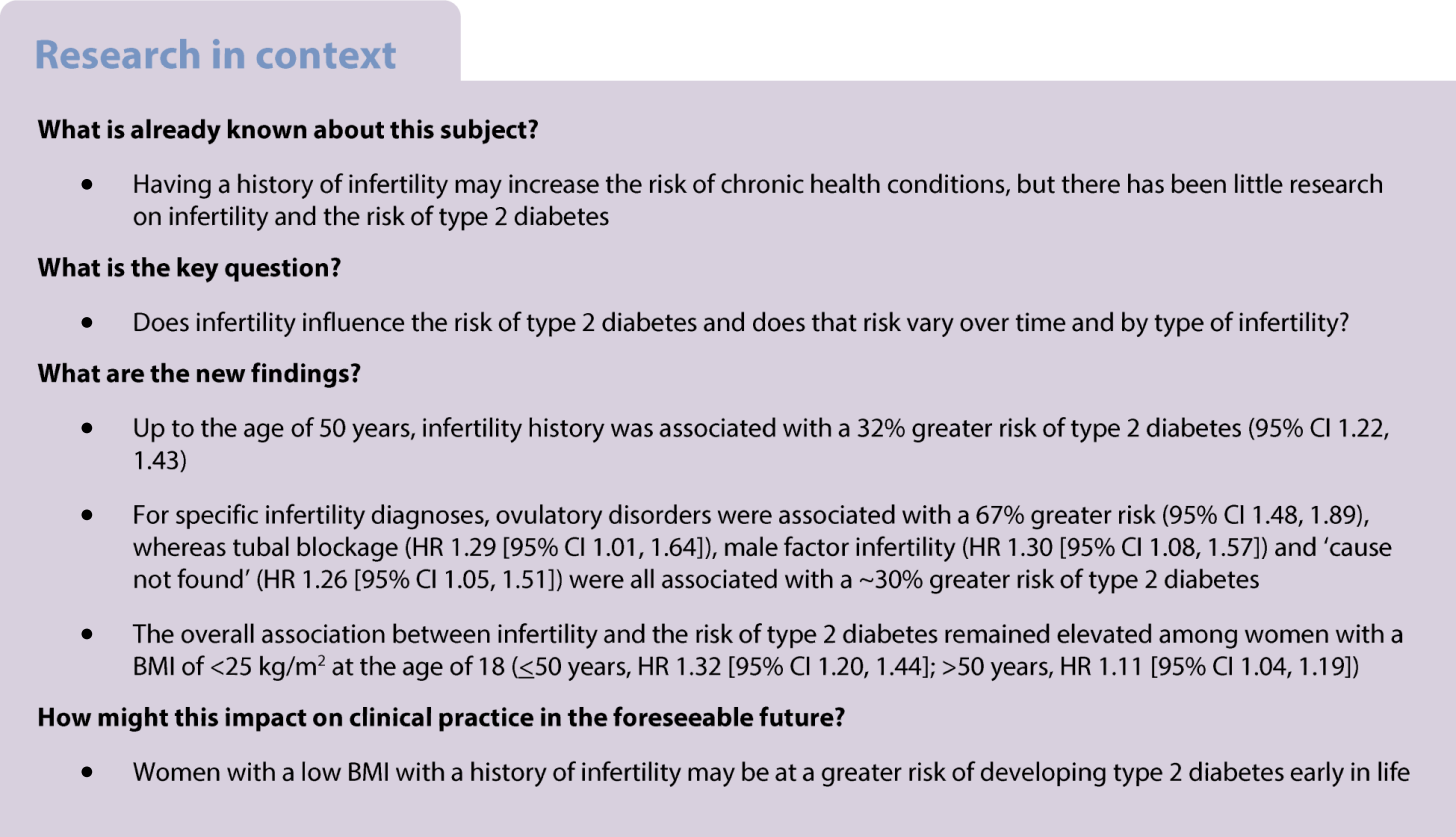


## Introduction

Infertility is estimated to burden between 8% and 16% of couples in the United States [1] and can be caused by a variety of factors, including underlying disease pathophysiology (e.g. endometriosis, polyendocrine metabolic ovarian syndrome [PMOS] and tubal infertility) and advanced maternal age. There is emerging research suggesting that infertility may represent an early-life window into future health and disease risk, as infertility has been observed to be associated with a greater risk of cardiometabolic disease [2–9] and mortality [10, 11].

There is evidence supporting the association between some of the causes of female infertility and the subsequent risk of cardiometabolic disease and type 2 diabetes. Previous research in the Nurses’ Health Study II (NHSII) found that women reporting a history of infertility (>12 months of trying to conceive) had a 20% greater risk of developing type 2 diabetes than those not reporting infertility (HR 1.20 [95% CI 1.14, 1.28]) [12]. This observation was also supported by findings from the Framingham Heart Study [13], the National Health and Nutrition Examination Survey [14], the Diabetes Prevention Program (DPP) [15] and the Optum administrative health claims [7]. Understanding whether infertility is an early-life marker of cardiometabolic vulnerability prior to type 2 diabetes diagnosis may shape targeted intervention strategies and screening behaviours in women. However, few studies have had a follow-up of sufficient duration to observe how risk changes across the life course or have been able to separately investigate infertility diagnoses individually, as prior research has observed associations between the risk of type 2 diabetes and PMOS [16], tubal factors [12] and endometriosis [17]. Furthermore, there is a paucity of research that has accounted for the influence of BMI as a shared risk factor for both infertility and type 2 diabetes.

We investigated the association between the history of infertility and the risk of type 2 diabetes among a cohort of women with approximately 30 years of longitudinal follow-up. We investigated the association between infertility history and blood levels of HbA_1c_ as an intermediate biomarker of type 2 diabetes risk. Given our prospective and longitudinal cohort, we investigated whether the association between infertility and type 2 diabetes varied by infertility diagnoses and age and whether the association between infertility and the risk of type 2 diabetes was independent of early-life body size, given the strong relationship between BMI and type 2 diabetes. We hypothesised that women with a history of infertility would have a higher risk of type 2 diabetes than those without infertility; specifically, women citing infertility due to tubal factors or ovulatory factors would have the highest risk of type 2 diabetes, compared with women without a history of infertility. We hypothesised that the association between infertility and type 2 diabetes would be stronger among younger ages and that the risk would be consistent among women with a low BMI.

## Methods

### Study population

Participants were enrolled in the NHSII in 1989, when 116,429 registered nurses between the ages of 25 and 42 years returned a mailed questionnaire. As described elsewhere [18], participants have returned questionnaires every 2 years subsequently, which collected a variety of information on chronic conditions and modifiable lifestyle factors and risk factors. In total, 116,429 women initially enrolled in the NHSII. For the main analysis, we excluded women who reported a diagnosis of diabetes (type 1 or type 2), stroke, myocardial infarction or coronary artery bypass grafting prior to June 1989. The main analyses compared women with a history of infertility with gravid women with no history of infertility. Thus, nulligravid women without a history of infertility were excluded. This study was approved by the Institutional Review Board of Brigham and Women’s Hospital and Michigan State University.

### Infertility

Between 1989 and 2001, participants self-reported on every questionnaire whether they had ‘tried to become pregnant for more than 1 year without success’, which is a standard definition of infertility for epidemiological studies [19]. After 2001 (when participants were aged 37 to 54 years), participants self-reported infertility on every other questionnaire until 2009, when participants were on average 45–62 years old*.* We classified participants as having experienced infertility if they indicated that they had tried to conceive for 1 year or more without success at any time until participants were 45 years old [10]. Participants were also asked to report the cause of their infertility and could choose all that applied from the following list: tubal blockage, ovulatory disorder, endometriosis, cervical mucus factor, male factor infertility, not investigated, not found and/or other. Participants in the NHSII are able to validly recall their infertility diagnoses: ovulatory infertility (93% concordance with the supplemental questionnaire and 95% concordance with medical records) [20] or endometriosis (97% concordance with medical records) [21].

### Type 2 diabetes

Participants self-reported physician-diagnosed type 2 diabetes on each questionnaire, and diagnoses were then medically confirmed by in-house research staff. Specifically, participants who reported having been diagnosed with type 2 diabetes were sent a supplemental questionnaire to confirm if they met the National Diabetes Data Group classification [22]: at least one classic symptom (excessive thirst, polyuria, unintentional weight loss or hunger) and a fasting plasma glucose concentration of ≥7.8 mmol/l (140 mg/dl) or a random plasma glucose concentration of ≥11.1 mmol/l (200 mg/dl); no symptoms but at least a twofold elevation in their plasma glucose concentration on more than one occasion (fasting ≥7.8 mmol/l, random ≥11.1 mmol/l, 2 h OGTT ≥11.1 mmol/l); or hypoglycaemic medication use (insulin or oral hypoglycaemic agent). In 1998, the criteria changed to adopt a new diagnostic threshold: a fasting plasma glucose concentration of ≥7.0 mmol/l (126 mg/dl) [23]. The Nurses’ Health Study cohort reported a high validity of this technique (98% concordance with medical records) [24]*.* Type 2 diabetes diagnoses have been confirmed until 2019.

### HbA_1c_

Establishment of the nested blood cohort has been described previously [25]. In brief, blood samples were collected between 1996 and 1999 from 29,611 NHSII cohort members who were aged 32–54 years at blood collection. Samples were shipped with an ice pack via overnight courier to the Harvard Cohorts’ biorepository, where they were processed and have been stored in continuously monitored liquid nitrogen freezers since collection. Participants completed a blood-draw questionnaire that recorded the date and time of blood collection and information on their current weight, parity, smoking status, alcohol and medication use, hours since last food intake and recent changes in menstrual cycle characteristics, as well as the first day of the menstrual cycle in which the blood samples were drawn.

HbA_1c_ reflects glycaemic status in the past 2–3 months. HbA_1c_ (%) was measured by turbidometric immunoassay in red blood cells using the Hitachi 911 Analyzer (Roche Diagnostics, Indianapolis, Indiana). For this analysis, we used existing HbA_1c_ data from several NHSII sub-studies (*n*=2388) on alcohol use, ischaemic stroke, diabetes and heart attack; all samples were assayed randomly independent of case/control status. All projects included 10% blinded quality control samples along with tested samples; the intra-assay coefficients of variation were less than 5% for HbA_1c_ [26, 27]. In sensitivity analyses, we excluded individuals with type 2 diabetes from previous case–control studies.

### Statistical analysis

Cox proportional hazard models were used to estimate the association between infertility history and the risk of type 2 diabetes. To account for the influence of age on type 2 diabetes risk, namely that the risk of type 2 diabetes increases with age and the number of susceptible people decreases with age, and to better take into account the influence of time since infertility, effect estimates were calculated among women ≤50 and >50 years of age, as has been done in other studies of infertility and type 2 diabetes [15]. The proportional hazard assumptions were tested by using an interaction term of age with the exposure variable, and met in the age-stratified models. Models for the overall association between infertility and type 2 diabetes did not meet the assumptions of proportional hazards and are not shown. Our main analysis investigated the association between ever having experienced infertility and the risk of incident type 2 diabetes. All multivariable models were adjusted for age (months) and calendar time (model 1). Model 2 also adjusted for BMI at the age of 18 years (17 categories), age at menarche (≤11, 12, 13 or 14+ years), marital status (never or ever/currently married), race (Black, Asian or other vs White), total breastfeeding duration (<3, 3–12 or >12 months), gravidity (0–1, 2, 3 or 4+ pregnancies), oral contraceptive use history (current, past or never), Alternative Healthy Eating Index (AHEI) 2010 diet quality score (in quintiles), menopausal status (premenopausal, postmenopausal or dubious/unsure/unknown), physical activity (0.1–1.0, 1.1–2.4, 2.5–5.9 or 6.0+ MET hours/week) and smoking status (never, former/past or current). Finally, model 3 adjusted for time-varying BMI + BMI^2^, which allowed information on BMI to vary every 2 years (continuous). Model 3 may represent associations with type 2 diabetes independent of adiposity later in life. In secondary analyses, we investigated differences by non-mutually exclusive self-reported causes of infertility (adjusting for model 3), age at first experiencing infertility (≤25, 26–30 or >30 years) and primary vs secondary fertility (the latter being infertility occurring after the birth of a previous child) (adjusting for model 3). Due to the possibility of confounding and effect modification by BMI, in addition to adjustment in multivariable models, we conducted a sensitivity analysis restricted to those who had a BMI of ≤25 kg/m^2^ at the age of 18 years, a time point that presumably precedes infertility and type 2 diabetes diagnoses.

For our HbA_1c_ analysis, we log-transformed HbA_1c_ for analysis to improve the normality of the distribution. We used linear regression models to estimate the associations between the history of infertility and HbA_1c_ with effect estimates presented as the per cent difference ([(exp (β) – 1) × 100]) with corresponding 95% CIs comparing women with a history of infertility with gravid women with no history of infertility. Batch-to-batch variability was accounted for using previously described regression methods [28].

## Results

At baseline, 21,085 women (22.2%) reported having experienced infertility. During 2,680,543 person-years of follow-up, 8638 women were diagnosed with type 2 diabetes. Women with a history of infertility were slightly more likely than women without a history of infertility to have a BMI of ≥30 kg/m^2^ at cohort baseline (13% vs 11%), have a BMI of ≥25 kg/m^2^ at the age of 18 (10% vs 9%), have an earlier age (≤11 years) at menarche (27% vs 24%) and be current smokers (14% vs 13%) (Table 1). They were also more likely than women without a history of infertility to be past oral contraceptive users (81% vs 76%) and be nulliparous at baseline (29% vs 12%). Among those with a history of infertility, the most commonly cited causes were ovulatory disorder (28%), male factor infertility (16%), ‘cause not investigated’ (20%) and ‘cause not found’ (16%).

**Table 1:** Age-standardised characteristics of the NHSII population at the 1989 baseline stratified by infertility history

Characteristic	History of infertility	
	Yes (*n*=21,085)	No (*n*=73,720)
Age, years, mean (SD)^a^	36.1 (4.1)	35.1 (4.5)
BMI, kg/m^2^, %		
<18.5	3.2	3.0
18.5 to <22.5	42.0	43.9
22.5 to <25	22.2	23.3
25 to <30	19.2	19.3
30+	13.4	10.6
BMI at the age of 18, kg/m^2^, %		
<18.5	16.2	14.2
18.5 to <22.5	60.9	63.2
22.5 to <25	13.0	14.0
25+	9.9	8.5
Age at menarche, years, %		
≤11	26.9	23.9
12–13	55.5	58.5
14+	17.6	17.6
Smoking status, %		
Never	64.0	63.9
Past	22.0	22.7
Current	14.0	13.4
Total physical activity, MET hours/week, %		
0 to <3	15.7	15.9
3 to <9	24.1	23.1
9 to <18	21.3	21.3
18 to <27	12.8	13.1
27 to <42	11.6	11.6
42+	14.5	15.0
Oral contraceptive use, %		
Current	5.0	9.5
Past	81.4	76.4
Never	13.7	14.1
Infertility diagnosis, %		
Ovulatory disorder	28.0	0.0
Endometriosis	10.5	0.0
Cervical mucus disorder	4.9	0.0
Tubal blockage	9.3	0.0
Male factor infertility	16.4	0.0
Cause not investigated	20.3	0.0
Cause not found	16.3	0.0
Other cause	11.6	0.0
Parity history, %		
Nulliparous	28.9	11.8
1	25.5	22.2
2	30.9	42.2
3+	14.8	23.8

Values are standardised to the age at enrolment (baseline) distribution of the study population. Values of polytomous variables may not sum to 100% due to rounding

^a^ Value is not age adjusted

The association between the history of infertility and the risk of type 2 diabetes differed before and after the age of 50 (*p* value, test for interaction: 0.0004). Overall, among women ≤50 years old, we observed that a history of infertility was associated with a 40% greater risk of type 2 diabetes (95% CI 1.29, 1.51) (Table 2, model 2). In the model that was independent of BMI changes later in life, infertility was associated with a 32% greater risk of type 2 diabetes (95% CI 1.22, 1.43) (Table 2, model 3). When specific infertility diagnoses were investigated separately, ovulatory disorder was associated with a 67% greater risk of type 2 diabetes (95% CI 1.48, 1.89), whereas tubal blockage (HR 1.29 [95% CI 1.01, 1.64]), male factor infertility (HR 1.30 [95% CI 1.08, 1.57]) and ‘cause not found’ (HR 1.26 [95% CI 1.05, 1.51]) were all associated with approximately a 30% greater risk of type 2 diabetes among women aged ≤50 years (Table 3).

**Table 2:** Association between a history of infertility and the risk of incident type 2 diabetes in the NHSII cohort from 1989 to 2019, stratified by participant age

History of infertility	*n*/person-years	HR for incident type 2 diabetes (95% CI)		
		Model 1	Model 2	Model 3
≤50 years old				
No	2129/1,171,074	1.0 (referent)	1.0 (referent)	1.0 (referent)
Yes	975/348,476	1.53 (1.41, 1.65)	1.40 (1.29, 1.51)	1.32 (1.22, 1.43)
>50 years old				
No	4094/899,151	1.0 (referent)	1.0 (referent)	1.0 (referent)
Yes	1440/261,842	1.20 (1.13, 1.28)	1.16 (1.09, 1.24)	1.12 (1.06, 1.19)

Model 1: Cox proportional hazards model adjusted for age (months) and calendar time

Model 2: additionally adjusted for BMI at the age of 18 (continuous), age at menarche (≤11, 12, 13 or 14+ years), marital status (never or ever/currently married), race (Black, Asian or other vs White), total breastfeeding duration (<3, 3–12 or >12 months), gravidity (0–1, 2, 3 or 4+ pregnancies), oral contraceptive use history (current, past or never), AHEI 2010 diet quality score (in quintiles), menopausal status (premenopausal, postmenopausal or dubious/unsure/unknown), physical activity (in quintiles [MET hours/week]) and smoking status (never, former/past or current)

Model 3: additionally adjusted for current BMI + BMI^2^ (continuous)

**Table 3:** Association between infertility diagnoses and the risk of incident type 2 diabetes in US women in the NHSII cohort from 1989 to 2019, stratified by participant age

Infertility diagnosis	*n*/person-years	HR for incident type 2 diabetes (95% CI)
≤50 years old		
Ovulatory disorder		
No	2129/1,171,074	1.0 (referent)
Yes	423/105,150	1.67 (1.48, 1.89)
Endometriosis		
No	2129/1,171,074	1.0 (referent)
Yes	70/42,123	0.89 (0.68, 1.17)
Cervical mucus disorder		
No	2129/1,171,074	1.0 (referent)
Yes	37/18,439	1.11 (0.76, 1.61)
Tubal blockage		
No	2129/1,171,074	1.0 (referent)
Yes	91/34,139	1.29 (1.01, 1.64)
Male factor infertility		
No	2129/1,171,074	1.0 (referent)
Yes	170/61,722	1.30 (1.08, 1.57)
Not investigated		
No	2129/1,171,074	1.0 (referent)
Yes	183/69,248	1.11 (0.95, 1.29)
Not found		
No	2129/1,171,074	1.0 (referent)
Yes	124/55,050	1.26 (1.05, 1.51)
Other reason		
No	2129/1,171,074	1.0 (referent)
Yes	117/48,395	1.06 (0.85, 1.32)
>50 years old		
Ovulatory disorder		
No	4094/899,151	1.0 (referent)
Yes	431/70,643	1.18 (1.05, 1.32)
Endometriosis		
No	4094/899,151	1.0 (referent)
Yes	171/32,127	1.17 (0.98, 1.40)
Cervical mucus disorder		
No	4094/899,151	1.0 (referent)
Yes	58/12,926	0.99 (0.74, 1.33)
Tubal blockage		
No	4094/899,151	1.0 (referent)
Yes	156/25,912	1.26 (1.05, 1.51)
Male factor infertility		
No	4094/899,151	1.0 (referent)
Yes	214/44,547	0.96 (0.82, 1.13)
Not investigated		
No	4094/899,151	1.0 (referent)
Yes	303/53,350	1.08 (0.96, 1.22)
Not found		
No	4094/899,151	1.0 (referent)
Yes	233/43,987	1.14 (0.99, 1.30)
Other reason		
No	4094/899,151	1.0 (referent)
Yes	182/35,166	1.07 (0.90, 1.26)

Multivariable adjusted model 3: adjusted for age (months) and calendar time, BMI at the age of 18 (continuous), age at menarche (≤11, 12, 13 or 14+ years), marital status (never or ever/currently married), race (Black, Asian or other vs White), total breastfeeding duration (<3, 3–12 or >12 months), gravidity (0–1, 2, 3 or 4+ pregnancies), oral contraceptive use history (current, past or never), AHEI 2010 diet quality score (in quintiles), menopausal status (premenopausal, postmenopausal or dubious/unsure/unknown), physical activity (in quintiles [MET hours/week]), smoking status (never, former/past or current) and BMI + BMI^2^ current (continuous)

Among women >50 years old, the association between infertility and type 2 diabetes attenuated and was associated with a 12% greater risk of type 2 diabetes overall in the models independent of BMI change (95% CI 1.06, 1.19) (Table 2). When specific infertility diagnoses were investigated, the associations were attenuated compared with associations at an earlier age; the HR for ovulatory disorders (HR 1.18 [95% CI 1.05, 1.32]) remained statistically significant, while, while the associations with male factor infertility (HR 0.96 [95% CI 0.82, 1.13]) and ‘cause not found’ (HR 1.14 [95% CI 0.99, 1.30]) did not. The association between tubal blockage and the risk of type 2 diabetes remained similar among women >50 years old to that in those ≤50 years old (HR 1.26 [95% CI 1.05, 1.51]) (Table 3).

When results were restricted to those with a BMI of <25 kg/m^2^ at the age of 18, the overall association between infertility and the risk of type 2 diabetes remained consistently elevated (≤50 years, HR 1.32 [95% CI 1.20, 1.44]; >50 years, HR 1.11 [95% CI 1.04, 1.19]) (Table 4). We did not observe any meaningful differences by age of the first report of infertility (electronic supplementary material [ESM] Table 1) or between primary and secondary infertility (ESM Table 2).

**Table 4:** Association between a history of infertility and the risk of incident type 2 diabetes in US women in the NHSII cohort from 1989 to 2019, restricted to participants with a BMI of <25 kg/m^2^ at the age of 18

History of infertility	*n*/person-years	HR for incident type 2 diabetes (95% CI)		
		Model 1	Model 2	Model 3
≤50 years old				
No	1561/1,075,015	1.0 (referent)	1.0 (referent)	1.0 (referent)
Yes	675/314,674	1.47 (1.34, 1.61)	1.42 (1.30, 1.56)	1.32 (1.20, 1.44)
>50 years old				
No	3533/831,154	1.0 (referent)	1.0 (referent)	1.0 (referent)
Yes	1214/239,680	1.19 (1.11, 1.27)	1.17 (1.09, 1.25)	1.11 (1.04, 1.19)

Model 1: adjusted for age (months) and calendar time

Model 2: additionally adjusted for BMI at the age of 18 (continuous), age at menarche (≤11, 12, 13 or 14+ years), marital status (never or ever/currently married), race (Black, Asian or other vs White), total breastfeeding duration (<3, 3–12 or >12 months), gravidity (0–1, 2, 3 or 4+ pregnancies), oral contraceptive use history (current, past or never), AHEI 2010 diet quality score (in quintiles), menopausal status (premenopausal, postmenopausal or dubious/unsure/unknown), physical activity (in quintiles [MET hours/week]) and smoking status (never, former/past or current)

Model 3: additionally adjusted for current BMI + BMI^2^ (continuous)

On average, women were aged 44 at the blood draw. We observed that, compared with women without a history of infertility, those with a history of infertility had slightly higher relative HbA_1c_ levels (relative difference: 0.68% [95% CI 0.04, 1.32]), reflecting a minimal absolute difference in HbA_1c_ for women with infertility (mean [SD]: 5.35% [0.43]) compared with women without infertility (mean [SD]: 5.30% [0.38]) (Table 5).

**Table 5:** Association between overall infertility history and blood levels of HbA_1c_ within the NHSII cohort (*N*=2399)

History of infertility	*n*	%, mean (SD)	Model 1	Model 2	Model 3
			Relative % difference (95% CI)^a^		
No^b^	1794	5.30 (0.38)	Referent	Referent	Referent
Yes	594	5.35 (0.43)	0.64 (−0.02, 1.29)	0.73 (0.08, 1.39)	0.68 (0.04, 1.32)

Model 1: adjusted for age at blood collection and batch-adjusted using all covariates from model 3

Model 2: additionally adjusted for BMI at the age of 18 (continuous), age at menarche (≤11, 12, 13 or 14+), marital status (never or ever/currently married), race (Black, Asian or other vs White), total breastfeeding duration (<3, 3–12 or >12 months), gravidity (0–1, 2, 3 or 4+ pregnancies), oral contraceptive use history (current, past or never), fasting status (dichotomous), time of blood collection (four categories), season of blood collection (winter, spring, autumn or summer) and luteal day (four categories)

Model 3: additionally adjusted for current BMI + BMI^2^ (continuous), AHEI 2010 diet quality score (in quintiles), menopausal status (premenopausal, postmenopausal or dubious/unsure/unknown), physical activity (in quintiles [MET hours/week]) and smoking status (never, current or former)

^a^ For log-transformed mean values of HbA_1c_, the difference in the biomarker was calculated with output from a general linear regression model and back transformed using ([exp(β) – 1] × 100). Extreme studentised deviate (ESD) outlying values were removed at the 0.05 level of statistical significance

^b^ The comparison group was gravid women who never reported infertility

## Discussion

Overall, we observed that women with a history of infertility, compared with gravid women without infertility, had a higher risk of developing type 2 diabetes, especially before the age of 51. This finding was consistent when restricted to women who had a low BMI at the age of 18. Specific infertility diagnoses that were associated with an elevated risk of type 2 diabetes included ovulatory disorders, tubal factors, male factor infertility and ‘cause not found’. Furthermore, women with a history of infertility had slightly higher relative levels of HbA_1c_ at midlife (on average at the age of 44).

Overall, our results are consistent with the majority of previous findings from our research group and others. Research using over 3 million health records from the Optum de-identified Cliniformatics Data Mart database between 2003 and 2016 observed a 44% greater risk of type 2 diabetes among infertile individuals than in non-infertile individuals; however, that study was only able to undertake a follow-up of participants for a mean of 4 years [7]. Findings from the DPP also observed that parous women with a history of infertility had an 80% higher risk of premenopausal type 2 diabetes than women without a history of infertility, and that attenuated to 63% among nulligravid women with a history of infertility, but the statistical power was limited [15]. Previous data from the NHSII with a shorter duration of follow-up revealed a 20% greater risk of type 2 diabetes among women with a history of infertility than women without a history of infertility [12]. By contrast, among over 17,000 Dutch women in the Prospect-European Prospective Investigation into Cancer and Nutrition (EPIC) cohort, consulting a physician for fertility problems was not associated with greater risk of diabetes after, on average, 9 years of follow-up [29]; however, the definition of infertility, the length of follow-up and differing population demographic and risk factors may have led to conflicting results. There is a larger body of literature that has investigated the association between parity and nulliparity and the risk of type 2 diabetes; however, these findings have also been conflicting [30, 31], possibly due to changes in adiposity and the gestational diabetes risk conferred with increasing parity.

To our knowledge, very few prior studies that have investigated overall infertility have also been able to separately investigate multiple infertility diagnoses. However, studies focused on underlying causes for infertility and the risk of type 2 diabetes are more common. The association between PMOS and cardiometabolic conditions, including type 2 diabetes, has been well documented [16, 32–43]; therefore, associations between ovulatory infertility and type 2 diabetes are presumed to be driven by PMOS, which is the most common pathology within this subtype. Ovulatory disorders (PMOS), tubal factors, male factor infertility and ‘reason not found’ have been observed in prior literature to be associated with the risk of type 2 diabetes [12]. We observed that, among women ≤50 years old, ovulatory disorders conferred the highest risk, while tubal factors, male factor infertility and ‘cause not found’ all conferred a similarly modest risk. Among women >50 years old, most associations weakened considerably. Among women >50 years old, there may be a decrease in the number of susceptible people, and there is a longer duration between the time of infertility and diabetes onset, which may influence risk. The association between ovulatory infertility and type 2 diabetes is consistent with other studies’ findings on a greater risk of type 2 diabetes among women with PMOS [16, 32–43]. The modest finding with tubal factor infertility may be driven by several mechanisms, including systemic inflammation caused by untreated sexually transmitted infections, which may lead to both tubal factor infertility and a higher risk of type 2 diabetes; severity of infertility; and residual confounding. Prior research has also observed an association between male factor infertility and the risk of type 2 diabetes (HR 1.15) [12]. There are several hypothesised mechanisms through which male factor infertility may be associated with type 2 diabetes: (1) couples who receive a diagnosis of male factor infertility may have experienced a longer time to pregnancy or a later age at first pregnancy; (2) receiving a diagnosis of male factor infertility may indicate that the couple has greater access to medical care; and (3) there may be residual confounding by paternal BMI or eating patterns that increases the risk of male factor infertility, which may also influence the female partner and may not be adequately captured in the current data.

While many studies have investigated insulin resistance measures as risk factors for infertility [44–46], few studies have investigated HbA_1c_ levels for women with a history of infertility later in life. The DPP observed similar levels of HbA_1c_ across infertile and parous groups [15]; however, this was among a population at high risk of developing type 2 diabetes and the time between infertility experience and HbA_1c_ measurement was not reported. Research from the National Health and Nutrition Examination Survey (NHANES), a cross-sectional study, observed that for every 1% increase in HbA_1c_, there was a 40% increase in the risk of reporting a history of infertility [47], but again the time between infertility experience and HbA_1c_ measurement was not reported. A causal association between HbA_1c_ and infertility has also been observed in studies employing Mendelian randomisation [47], suggesting that HbA_1c_ may influence infertility risk [44]. We did not observe a strong or clinically meaningful association between infertility history and HbA_1c_ levels later in life (with an average age of 44 at the blood draw).

Our findings are in agreement with three prior studies [7, 12, 15], which observed that a history of infertility was associated with a greater risk of type 2 diabetes later in life. This suggests that infertility history, especially a history of ovulatory infertility, may serve as an early-life marker of metabolic risk. A critical next step in this work is identifying interventions or screening practices for women with infertility to improve their risk of type 2 diabetes. There are several possible points of intervention that should be further investigated among women with infertility. For example, among women who are using infertility care, the impact of measuring glycaemic status as part of infertility should be further investigated [45], as this may be an opportunity for early screening and intervention. Additionally, information on infertility history should be a part of an individual’s medical chart and, among women who have a history of infertility, future research should investigate the effectiveness of early screening for diabetes, in addition to lifestyle modifications to reduce the risk of type 2 diabetes. While there is increased provider awareness that understanding a woman’s pregnancy history has important health implications for cardiometabolic health later in life [48], infertility should also be considered as an early-life marker of cardiometabolic risk.

This study has many strengths, including its large sample size, detailed information on infertility (e.g. infertility diagnoses, age at infertility, and primary vs secondary infertility), time-varying covariate data and over 30 years of follow-up. However, there are also some limitations that must be considered. Infertility history was based on self-report and to be classified as infertile a participant had to have been trying to achieve pregnancy and have not conceived for at least 12 months. There are participants who may never have tried to become pregnant and, thus, were not at risk of experiencing or recognising infertility. We attempted to exclude these individuals from our comparison group by restricting our comparison population to participants who achieved pregnancy and who never reported infertility. While we may expect some misclassification of self-reported infertility, prior research has shown a high accuracy of recall of infertility diagnoses [20, 21], and we would expect that any misclassification would be non-differential with respect to type 2 diabetes history, driving the primary findings towards the null. Unfortunately, our questionnaire is unable to disentangle PMOS from ovulatory infertility. Participants in this cohort are not a random sample of all women in the USA; however, it is not likely that the biological associations observed in this cohort would substantially differ from US women. Moreover, the medical background of this population reduces confounding by socioeconomic status and education and reduces possible misclassification of self-reported medical history, both of which enhance the internal validity of the study. Although this analysis did not incorporate information on fertility treatment, recent meta-analyses have not observed associations between fertility treatment and the risk of type 2 diabetes [49].

In conclusion, we observed that participants who had experienced infertility had a greater risk of developing type 2 diabetes than participants who had never experienced infertility. This association was strongest at younger ages (e.g. ≤50 years old) and for those who had experienced infertility caused by ovulatory disorders, tubal factor infertility or male factor infertility and for those for whom the cause of their infertility was not found. Infertility may be an important indicator of a heightened risk of type 2 diabetes during reproductive years, potentially allowing more time for intervention and lifestyle changes to reduce the type 2 diabetes risk in midlife.

## Supplementary Information

Below is the link to the electronic supplementary material.

ESM Tables (PDF 94 KB)

## Data Availability

Data regarding any of the participants in the study have not been previously published unless specified. Because of participant confidentiality and privacy concerns, data cannot be shared publicly. According to standardised controlled access procedures, requests to access NHSII data must be submitted in writing. Applications to use NHSII resources will be reviewed by our External Collaborations Committee to verify that the proposed use maintains the protection of the privacy of participants and the confidentiality of the data. Investigators wishing to use NHSII data can find instructions for access at https://www.nurseshealthstudy.org/researchers (contact email: nhsaccess@channing.harvard.edu), which includes submission of a brief description of the investigators’ proposed project aim.

## Abbreviations

AHEI: Alternative Healthy Eating Index
DPP: Diabetes Prevention Program
NHSII: Nurses’ Health Study II
PMOS: Polyendocrine metabolic ovarian syndrome
