# Supplementary material for: History of infertility, risk of type 2 diabetes and HbA1c levels in the Nurses’ Health Study II
Source: Diabetologia. 2026 Jun 30;69(9):2523–33. doi: 10.1007/s00125-026-06784-5 (PMC13424155; doi:10.1007/s00125-026-06784-5)
Supplement: Supplementary file 1 — ESM Tables (PDF 94 KB) [file 125_2026_6784_MOESM1_ESM.pdf]

ESM Table 1. Association between age at first reported overall (all-cause) infertility and the risk of incident type 2 diabetes in US women in the Nurses' Health Study II cohort from 1989 to 2019. Stratified by participant age less than or equal to 50 years old

		Hazard Ratio for Type 2 Diabetes (95% CI)		
Age at first reported infertility	Without history of infertility	Age ≤ 25	Age 26-30	Age >30
		≤ 50 years old		
Yes	1.0 (Referent)	1.33 (1.19, 1.48)	1.32 (1.17, 1.49)	1.30 (1.14, 1.49)
		> 50 years old		
Yes	1.0 (Referent)	1.14 (1.05, 1.24)	1.09 (0.99, 1.20)	1.13 (1.00, 1.27)

Multivariable adjusted model 3: Adjusted for age (months) and calendar time, BMI at age 18 (continuous), age at menarche <11, 12, 13, 14+), marital status (never, ever/currently married), race (non-white, white), total breastfeeding duration (<3, 3-12, >12 months), gravidity (0-1, 2, 3, 4+), oral contraceptive use history (current, past, never), AHEI 2010 diet quality score (in quintiles), menopausal status (pre, post, dubious/unsure/unknown), physical activity (in quintiles, MET hours/week), smoking status (never, former/past, current), BMI + BMI^2 current (continuous)

ESM Table 2. Association between primary and secondary infertility and the risk of incident type 2 diabetes in US women in the Nurses' Health Study II cohort from 1989 to 2019. Stratified by participant age less than or equal to 50 years old

**History of Infertility**	Hazard Ratio for Type 2 Diabetes	
	Primary Infertility	Secondary Infertility
	≤ 50 years old	
No	1.0 (Referent)	1.0 (Referent)
Yes	1.36 (1.24, 1.50)	1.24 (1.10, 1.40)
	> 50 years old	
No	1.0 (Referent)	1.0 (Referent)
Yes	1.11 (1.03, 1.19)	1.15 (1.05, 1.26)

Multivariable adjusted model: Adjusted for age (months) and calendar time, BMI at age 18 (continuous), age at menarche <11, 12, 13, 14+), marital status (never, ever/currently married), race (non-white, white), total breastfeeding duration (<3, 3-12, >12 months), gravidity (0-1, 2, 3, 4+), oral contraceptive use history (current, past, never), AHEI 2010 diet quality score (in quintiles), menopausal status (pre, post, dubious/unsure/unknown), physical activity (in quintiles, MET hours/week), smoking status (never, former/past, current), BMI + BMI^2 current (continuous)
